# Efficacy and Tolerance of Vascular Electrical Stimulation Therapy in the Management of Vaso-Occlusive Crises in Patients with Sickle Cell Disease: A Phase II Single-Centre Randomized Study in Ivory Coast

**DOI:** 10.1155/2021/1373754

**Published:** 2021-02-04

**Authors:** Renée-Paule Botti, Sie Saïda Bokoum, Etienne L'Hermite, Dohoma Alexis Silue, Boidy Kouakou, Sarah Anastasie Bognini, Serge Arnaud Agoua, Edgar Mandeng Ma Linwa, Roméo Ayemou, Kouassi Gustave Koffi

**Affiliations:** ^1^Clinical Hematology Unit, CHU de Yopougon, BP 3709 Abidjan 08, Abidjan, Côte d'Ivoire; ^2^Diavein SAS, 227 Rte de La Chapelle, 74540 St Felix, France; ^3^Faculty of Health Sciences, University of Buea, Buea, Cameroon

## Abstract

**Background:**

Vaso-occlusive crisis (VOC) is the primary cause of hospitalization in patients with sickle cell disease. Treatment mainly consists of intravenous morphine or nonsteroidal anti-inflammatory drugs (NSAIDs), which have many dose-related side effects. The question arises as to whether vascular electrical stimulation therapy (VEST) could be effective or not on VOCs.

**Objective:**

To measure the effectiveness and safety of VEST in reducing the median time spent in severe VOC.

**Methods:**

We conducted a phase II, single blinded, randomized, controlled, triple-arm, comparative trial. We included thirty (30) adult patients with severe vaso-occlusive crisis. The study arms were divided as follows: our control group (group 0) constituted of 10 patients followed with conventional therapy (Analgesics + Hydration + NSAIDs), while 20 patients were divided equally into two interventional arms—10 patients followed with VEST + Analgesics + Hydration (group 1) and the other 10 patients followed with VEST + Analgesics + Hydration + NSAIDs (group 2). The primary efficacy endpoint was median time to severe crisis elimination. The secondary end points were median time to end-of-crisis, median tramadol consumption, progress of the haemoglobin level over 3 days, side effects, and treatment failure.

**Results:**

The age ranged from 14 to 37 years, including 23 women. We noted a beneficial influence of the VEST on the median time to severe crisis (VAS greater than 2) elimination; 17 hours (group 1) against 3.5 hours (group 2) *p*=0.0166 and 4 hours (group 3) with *p* value = 0.0448. Similar significant results were obtained on the diminution of total duration of the crisis (VAS over 0) and median tramadol consumption in patients in the interventional arms.

**Conclusion:**

These statistically significant results in the interventional arms suggest that VEST could be an alternative treatment of VOC in sickle cell patients.

## 1. Introduction

Sickle cell disease is the most common genetic haemoglobin disorder and most commonly affects individuals of African descent [1]. Ivory Coast is one of the fifteen most affected countries in the world [2].

Sickle cell disease or sickle cell anaemia is an autosomal recessive genetic disease involving the inheritance of abnormal haemoglobin. It is a point mutation of haemoglobin in which the glutamic acid in position 6 on the *β* chain is replaced by valine. The product of this autosomal mutation haemoglobin S is susceptible to intracellular polymerization under hypoxic conditions. These intracellular polymers increase the rigidity of erythrocytes, causing a distortion of their membrane that leads to the formation of weakened “sickle-shaped” red blood cells. These abnormal red blood cells are the root cause of the characteristic chronic anaemia in the disease [3]. One of the severe symptomatic manifestations of sickle cell disease is the occurrence of very intense and painful vaso-occlusive crises (VOCs), which are sometimes resistant to usual analgesics. Recent physiopathological data indicate a direct involvement of the vascular endothelium, multiple cellular interactions, and cellular activation processes that imply inflammatory mechanisms in the initiation and extension of vaso-occlusion [4]. Therefore, there are many factors that trigger and start VOCs. A combination of physiological characteristics related to sickle red cells and haemolysis results in both cell adhesion and vasoconstriction phenomena on both a prothrombotic and inflammatory level [4].

Numerous studies have been carried out with the aim of countering the causes of the crises, namely heparin for hypercoagulability [5] and nonsteroidal anti-inflammatory drugs (NSAIDs) for inflammation and vaso-occlusion [6–8]. Finally, the therapy involving antibodies directed against adhesion molecules (crizanlizumab) [9] has received its first global approval in the USA, where it is indicated to reduce the frequency of VOCs in adults and paediatric patients aged ≥16 years with sickle cell disease. The drug is also under regulatory review in the EU for the prevention of VOCs in patients with sickle cell disease.

Most of these studies have had no significant result. However, the use of antibodies appears promising, but the cost of treatment remains prohibitive for most patients. Vascular electrical stimulation therapy (VEST) has been investigated as a method for the prevention of haemodynamic and thromboembolic phenomena, dilation of veins and arteries, and acceleration of blood flow velocity and, thus, blood flow [10–12]. It is administered by a medical device using two or four electrodes placed in contact with the patients' skin. This device delivers electrical stimulation that engages the vascular smooth muscle as well as the endothelium via the adrenergic terminals. The delivered pulse has a duration of 2 to 3 ms and has a sinusoidal form upwards and an inverted sinusoidal form downwards. The typical voltage level is 25 V ± 5 V. The triggering and extension of a sickle cell VOC is multifactorial, combining rheological problems and vasoconstriction with a procoagulant and inflammatory environment. Our motivation for evaluating VEST in this context was based on the ability of this therapy to address 3 out of 4 of these factors: increased blood flow, vasodilation, and decreased platelet aggregation. Inflammation, the fourth factor, has not yet been identified as a process altered by VEST; we wanted to evaluate the effect of VEST treatment with and without NSAIDs in two separate arms, so as to be able to evaluate their potential synergy. For this reason, we decided to administer NSAIDs in the control arm. Given the low iatrogenic risk found in previous studies, it seemed appropriate to evaluate the tolerance and efficacy of VEST in managing VOCs in patients with sickle cell disease.

## 2. Materials and Methods

This was a phase II randomized, controlled, single-blinded, triple-arm clinical trial conducted at the Clinical Haematological Department of the Yopougon University Hospital, Abidjan, in Ivory Coast, over a 5-month period from June 2018 to October 2018.

Patients were eligible for inclusion if they had sickle cell disease type SS, SC, S*β*0, or S*β*+ documented by genotypic tests, were at least 14 years old, presented with VOC, and had a score of pain of at least 6 on the visual analogue scale (VAS) that had been progressing for more than 4 hours under analgesics and could not be attributed to another cause.

Exclusion criteria included patients with suspicion of acute chest syndrome, severe anaemia (Hb < 6 g/dL), or whose symptoms led to a suspicion of infection (temperature ≤35°C or >38.5°C, rising CRP over 3 days). The study also excluded patients with a pacemaker or known history of heart failure, women who were pregnant or breast feeding, patients with any psychological disorder, sociological conditions, or geographic locations that could hinder compliance with the study procedures or monitoring schedules.

### 2.1. Study Design

Patients with a major sickle cell disorder over the age of 14 presenting with severe VOCs and had a score of pain of 6 or more on the VAS were first treated using the department's emergency protocols with the appropriate hydration and analgesia. Patients treated this way were then offered to participate in the study after receiving the required regulatory information. An informed consent form was obtained from all study participants.

Patients were randomized (1 : 1 : 1) using a randomization chart to receive either VEST or not. The treatment schedule was as follows: conventional therapy group 0 (control group), with 10 patients under Analgesic + Hydration + NSAID; and two interventional arms including group 1, with 10 patients under VEST + Analgesic + Hydration, and group 2, with 10 patients under VEST + Analgesic + Hydration + NSAID. Patients in the interventional arms received VEST at clinical doses (little muscular tremors at the limbs) within 4 hours following their admission and for the subsequent two days if the pain persisted. Pain was said to be persistent if the VAS score was greater than 2. The NSAID used was ketoprofen at a dose of 1 mg/kg every 8 hours (maximum 300 mg/24 h). The procedure used to initiate VEST involved either placing two electrodes under each wrist (or palms) and 2 electrodes under each calf, or, one circular electrode around the torso and electrodes under each calf or two electrodes at the upper back (or upper chest) and 2 electrodes under each calf. VEST takes the form of an inverted sinusoidal pulse of 3 ms at 25 V peak on a frequency of 0.8 Hz alternated every 8 pulses.

Patients in the control group were connected to the stimulator without electrical impulses being administered. For ethical reasons, analgesics and intravenous hydration were maintained in all study arms.

### 2.2. Study Endpoints

The primary study endpoint was the time to severe crisis elimination (tSCE). Time to severe crisis elimination was defined as time spent with a score of pain greater than 2 on the VAS. This VAS value was chosen as the value from which a patient normally can manage their crisis with ambulatory oral analgesics.

The secondary endpoints were time to end-of-crisis, median consumption of tramadol, and progress of haemoglobin level over 3 days. The tolerability of treatment throughout the study was also evaluated. Time to end-of-crisis (tEC) was defined as time spent with a score of pain greater than 0 on the VAS.

Blood tests performed at admission and on day 3 of hospitalization included a thick blood smear, a complete blood count, and C-reactive protein (CRP) count. This was performed in order to exclude patients with infections (rising CRP) and/or severe anaemia and also record the progression of haemolysis.

### 2.3. Sample Size

Based on the available data, the intermediate endpoint (percentage reduction in median time to severe crisis elimination) was aimed at 50%. Meanwhile, the final endpoint (percentage reduction in time to end-of-crisis) was aimed at 25%.

The method proposed by [13] and that implemented by Parsons et al. [14, 15] in designing adaptive trials were used. These methods taking into account an overall alpha risk of 2.5% in unilateral configuration, a correlation of 0.8 between the intermediate and final endpoint, a complete follow-up of all patients to the final endpoint, inclusion in each arm of the 10-patient trial for interim analysis (total 30), then 30 in the last step (total 90, grand total for study of 120); a strength > 80% will be provided to reject the null hypothesis and conclude on the efficacy of an experimental arm.

### 2.4. Statistical Analysis

This was a time-to-event trial. The crises data (VAS > 2 and VAS > 0) were evaluated using the Kaplan–Meier method. The curves were compared by the Cox test for survival curve equality.

For each endpoint, a bivariate analysis was performed with all independent variables (VAS score on admission, phenotype, CRP, haemoglobin level, white blood cell count, and type of treatment administered). The analysis of the end-points was done with the Cox proportional hazards model coupled with the Breslow method.

A multivariate model was subsequently used to identify the independent factors associated with the different endpoints measured. The alpha risk was set at 5%.

### 2.5. Ethics and Consent to Participate

The study was approved by the National Ethics Committee for Health and Life Sciences (N 080-18/MSHP/CNESVS-km). Written informed consent was obtained from study participants.

## 3. Results

### 3.1. Patient Population

Out of the 40 patients received and evaluated to participate in the study, 36 patients met the criteria for the study and were enrolled. Four patients were later excluded for very high CRP levels on the third day and two patients for protocol deviation. Overall, the study included 30 patients divided into three groups of 10 patients each.

Patient baseline characteristics are presented in Table 1. Patients' ages ranged from 14 to 37 years old (median 19 years). Twenty-three (23) were women. Patients were homogeneously distributed between the three arms. In terms of genotypes, after randomization, there was an imbalance in the control group, which only had 1 patient with the SS genotype compared with three in group 1 and six in group 2. The most common pain location was the legs (19 patients) followed by the back (8 patients) and the arms (5 patients). The median VAS score at admission was 9.5 in the interventional groups and 10 in the control group.

### 3.2. Efficacy Data

**Figure 1:**
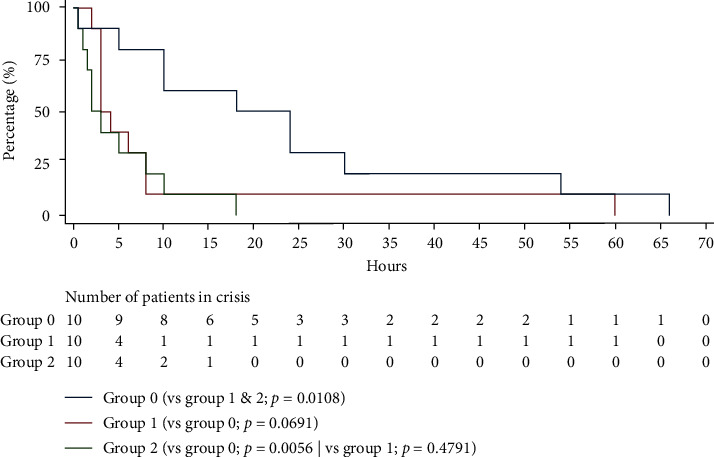
Severe crisis elimination time (VAS >2) by treatment (Cox test survival curve equality).

The tSCE was significantly decreased for the interventional groups combined (group 1 and 2) with a *p* value of 0.0108 (Figure 1). However, only group 2 had a significant reduction in tSCE compared with the control group (*p*=0.0056). Less than 25% of patients in severe crisis required up to 10 hours of treatment (vs 30 hours for the control group). All patients in group 2 emerged from a severe crisis within 20 hours of treatment (vs more than 65 hours for the control group).

**Figure 2:**
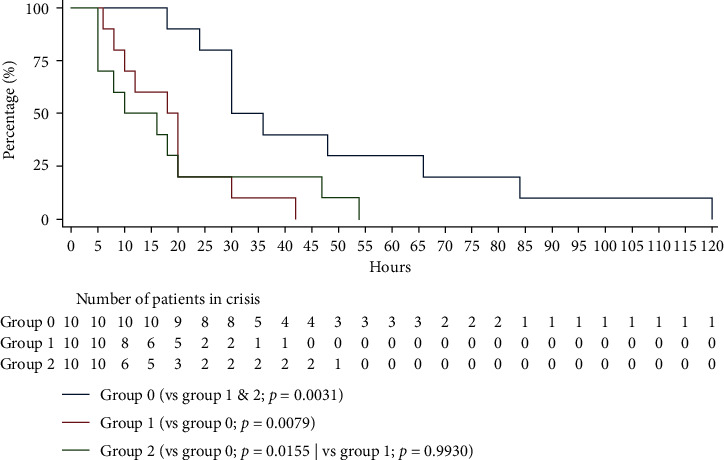
Time to end-of-crisis (tEC) by group of patients (Cox test survival curve equality).

This time was significantly decreased in the groups with VEST (group 1 and 2) compared with the control group, with a *p* value of 0.0031 (Figure 2). The tEC was significantly reduced in each VEST group compared with the control group (group 1, *p*=0.0079; group 2, *p*=0.0155). It took 20 hours of treatment for 75% of patients who received VEST (group 1 and group 2) to no longer be in crisis, compared with 66 hours for the control group. All patients in groups 1 and 2 emerged from a crisis within 42 hours and 54 hours of treatment, respectively, compared with 120 hours for the control group.

There was no significant difference in either tSCE (*p*=0.4791) and tEC (*p*=0.9930) between the two interventional arms. The tEC was also significantly lower in the interventional arms (VEST 19 hours, *p*=0.0079; VEST + NSAID 13 hours, *p*=0.0155) than in the control arm (33 hours). Other parameters evaluated in the univariate analysis are presented in Table 2. The tSCE and tEC were not significantly affected by phenotype, CRP, haemoglobin level, or white blood cell count at admission. The tSCE was, however, associated with pain intensity at admission.

The median consumption of tramadol (Table 3) was significantly lower in the interventional arms (VEST, *p*=0.0137; VEST + NSAIDs, *p*=0.0036) than in the control arm. The difference between the control group and combined VEST groups was also significant (*p*=0.0049), while the difference in median tramadol consumption between the two interventional arms was not significant (*p*=0.151). Progression of the haemoglobin level was not influenced by the type of treatment offered. This could imply that VEST does not increase risk of haemolysis and severe anaemia.

There was one adverse event reported during the study. One patient reported a minor paraesthesia-like side effect in the lower limbs at the end of stimulation, but the symptoms quickly decreased after treatment was stopped. No other adverse effects were reported.

In a multivariate analysis (Table 4), there was no correlation between tSCE or tEC and either CRP > 12 mg/L, VAS score, haemoglobin, or white blood cell levels at admission; however, there was a significant reduction in tSCE and tEC in the interventional groups compared with patients in the control group (*p* ≤ 0.007 for VEST and *p* < 0.001 for VEST + NSAID).

Male gender was also significantly associated with reduction in tEC (*p*=0.004), while the SS haemoglobin phenotype was significantly associated with reduction in tSCE (*p*=0.043).

## 4. Discussion

This phase II prospective, single-centre, study evaluated the efficacy of VEST in reducing the average time to severe crisis elimination (tSCE), i.e., the time spent by patients in crisis with a pain score greater than 2 on the VAS, compared with the current standard of care.

The absence of significant variation in the various sociodemographic and clinical parameters (age, sex, electrophoresis phenotype, location of pain, admission VAS, CRP, and blood count) for the control group and the interventional groups demonstrates that these 3 groups are homogeneous with respect to patient baseline characteristics and therefore can be compared. Also, the tSCE was not correlated with other independent variables such as the location of pain, sickle cell disease phenotype, CRP at admission, or blood count data.

The time to end-of-crisis (tEC), i.e., the time spent by patients in crisis with a pain score greater than 0 on the VAS, was also reduced in both interventional groups compared with the control group. Tramadol consumption was significantly higher in the control group compared with the interventional groups. It is therefore possible that the longer crisis duration in the control group combined with increased analgesic use would be expected to prolong duration of hospital stay and thus patient care cost. VEST did not seem to increase risk of haemolysis. On the other hand, the fact that the control group received the VEST treatment without the device being turned on and the small size of the sample were considered limitations of the study. Likewise, the lack of difference between groups 1 and 2, who were treated with VEST ± NSAIDs, could be explained by the small size of the sample. A second ongoing study with a larger sample could resolve this issue while commenting on an additional control group.

Therapeutic progress has for many years been based on the search for new treatments that not only circumscribe the effects of VOCs but which also control the underlying aetiology of these episodes, with the goal of reducing the duration of these painful and deleterious episodes in sickle cell patients. Advances in knowledge on the pathophysiology of VOCs have guided efforts to improve care, focusing on countering the factors intrinsically linked to the onset of VOCs. These are rheological disorders related to stress reticulocyte adhesion, blood hypercoagulability, and inflammation. On this basis, vasodilator drugs such as ginko biloba [3], inhaled nitric oxide [16], anticoagulants [5], and NSAIDs [6–8] have failed to demonstrate the ability to significantly reduce the duration or intensity of crises.

Among these studies, it is worth noting the reports from clinical trials involving TENS (transcutaneous electrical nerve stimulation). Literature review suggests that, in the assessment of pain on a scale of 1 to 10 after one hour and four hours, no difference was found between the TENS and “sham” TENS treatment groups. There was also no difference between the groups in the amount of analgesics used. Moreover, given the very low quality of the evidence, the authors could not conclude on the superiority of TENS in improving overall satisfaction compared with “sham” TENS [17].

Results from the aforementioned studies reflect the difficulty in significantly affecting the pathophysiological process involved, once a VOC has begun, through the correction of only one factor in a complex and interdependent, multifactorial physiological context.

VEST is a promising new therapy that is able to simultaneously address a multitude of factors involved in the development of VOCs and can do so at a relatively well-controlled level of iatrogenic risk. Indeed, the strength of VEST lies in the fact that it induces both mechanical effects allowing the improvement of the rheological parameters of the vascular system and also biological effects, with the main consequence being increased fibrinolytic action. The consequence of this multifactorial action is the rapid restoration of the deficient blood circulation in sickle cell patients in crisis and, consequently, a decrease in its symptomatic manifestation such as pain.

The hypothesized benefit of combining treatments that address several biological and rheological factors involved in VOCs is, however, questionable due to the lack of a significant difference obtained between the two interventional arms combining the use of VEST with or without NSAIDs as anti-inflammatory treatment. Indeed, based on the hypothesis that the VEST stimulation has no impact on the inflammatory process, we specifically evaluated the possible synergy of this mode of action through the use of NSAIDs in one of the interventional groups. The effectiveness of NSAIDs on the reduction of VOC has long been demonstrated [7]. The absence of a significant difference in the two interventional arms, in the reduction of VOC, suggests that VEST could constitute a therapeutic alternative in the management of VOC of sickle cell disease. To confirm this hypothesis, further studies are needed that evaluate the efficacy of vest in a larger population.

## 5. Conclusion

This study demonstrated a significant and clinically important reduction in time spent in severe crisis when patients received VEST and non-NSAID analgesics alone or in combination with NSAIDs compared with the current standard of care. The benefit of VEST demonstrated in this phase II study supports continued research into this novel therapy and a phase III study is ongoing (PACTR201907740118144). Further research activities are warranted to evaluate VEST technology in other countries of Central and West Africa, where there are significant disparities in the genotypic profiles of patients with sickle cell disease. This could lead to results that are significantly different from those obtained in Ivory Coast.

## Figures and Tables

**Table 1:** Baseline patient characteristics (age, sex, genotype, pain location, and VAS admission score).

Parameters	Placebo (*n* = 10)	VEST (*n* = 10)	NSAID + VEST (*n* = 10)	*p*
Median age, years (range)	9 (17–20)	27 (18–36)	23 (22–27)	0.0752 NS
Sex, *n* (%)				
Female	7 (70)	8 (80)	7 (70)	0.999 NS
Male	3 (30)	2 (20)	3 (30)	
Phenotype, *n* (%)				
SSFA2	1 (100)	0 (0)	0 (0)	
SFA2	7 (44)	6 (38)	3 (19)	0.185 NS
SC	1 (10)	3 (30)	6 (60)	
SAFA2	1 (33)	1 (33)	1 (33)	
Pain location, *n* (%)				
Arm	1 (20)	4 (80)	0 (0)	0.094 NS
Leg	7 (37)	5 (26)	7 (37)	0.709 NS
Back	2 (25)	2 (25)	4 (50)	0.668 NS
VAS Score at admission	10 (8–10)	9.5 (8–10)	9.5 (8–10)	0.5618 NS

NS, not significant; VAS, visual analogue score.

**Table 2:** Severe and total vaso-occlusive crisis elimination time by age, sex, VAS admission score, disease phenotype, haematology, and treatment arm.

Parameters	Severe crisis elimination time (time to VAS ≤ 2), h	*p*	Total crisis elimination time (time to VAS = 0), h	*p*
Age, years				
15–25	6.5 (2.5–15)	0.4526 NS		
25–35	4 (3–8)			
>35	14 (7–20)			
Sex				
Male	3.5 (2.5–12.5)	0.2894 NS		
Female	8 (3–16)			
VAS at admission				
6–9	3 (1.5–8)	**0.0110 S**	30 (16–42)	0.2938 NS
10	10 (5–20)		18 (10–24)	
Phenotype				
SSFA2	9 (5–20)	0.4199 NS	20 (18–47)	0.6304 NS
SFA2	3 (1–14)		8 (5–48)	
SC	4.5 (3–9)		20 (10–30)	
SAFA2	20 (20–20)		30 (30–30)	
CRP, mg/L				
≤12	5.5 (2.5–9)	0.1511 NS	20 (14–30)	0.8598 NS
>12	15 (3–30)		24 (8–47)	
Haemoglobin, g/dL				
<8	8 (3–20)	0.7443 NS	20 (10–30)	0.9359 NS
8–10	8 (3–14)		30 (6–48)	
>10	4.5 (3–10)		30 (30–30)	
White blood cell count, cells/mm^3^				
<10000	2.5 (1.25–8.5)	0.2985 NS	25 (12–54)	0.5833 NS
10000–15000	8 (3–20)		19 (3–33)	
>15000	6 (3–8)		30 (30–30)	
Treatment arm				
Group 0	17 (8–54)	**0.0374 S**	33 (30–66)	**0.0052 S**
Group 1	3.5 (3–8)		19 (10–20)	
Group 2	4 (1.5–10)		13 (5–20)	

CRP, C-reactive protein; h, hours; NS, not significant; VAS, visual analogue score; *p*, *p* value with significance set at less than 0.05. Bold values imply the difference observed is statistically significant.

**Table 3:** Consumption of tramadol according to various parameters (VAS at admission, phenotype, CRP at admission, haemoglobin, GR count, and treatment administered).

Parameters	Tramadol uptake	*p*
CRP		
<12	3 (2–4)	
≥12	4 (3–5)	0.1918 NS
Arms		
Group 0	5 (4–7)	**0.0049 S**
Group 1	3 (3–4)	
Group 2	2.5 (1–3)	

CRP, C-reactive protein; NS, not significant; VAS, visual analogue score; *p*, *p* value with significance set at less than 0.05 Bold values imply that the tramadol uptake was significantly different between the three study arms. *P* = 0,004 (<0.05).

**Table 4:** Multivariate analysis of severe crisis elimination time (time to VAS > 2) and total crisis elimination time (VAS = 0) (Cox proportional hazards model).

Variable	Severe crisis elimination time, h (VAS ≤ 2)		Total crisis elimination time, h (VAS = 0)	
	HR (95% CI)	*p*	HR (95% CI)	*p*
Male gender	1.53 (0.45–5.20)	0.491 NS	7.21 (1.89–27.48)	**0.004 S**
Age, years				
15–25	1		1	
25–35	0.38 (0.08–1.85)	0.232 NS	0.83 (0.16–4.40)	0.830 NS
>35	0.69 (0.07–6.89)	0.756 NS	0.99 (0.14–7.02)	<0.999 NS
VAS at h0 = 10 phenotype	0.31 (0.09–1.07)	0.064 NS	1.37 (0.46–4.08)	0.574 NS
SAFA2	1		1	
SC	0.21 (0.01–5.71)	0.354 NS	1.75 (0.07–44.47)	0.734 NS
SS	0.03 (0.01–2.08)	**0.043 S**	0.20 (0.01–10.03)	0.419 NS
SFA2	0.10 (0.01–9.22)	0.324 NS	2.73 (0.04–198.43	0.646 NS
CRP > 12 Hb	1.05 (0.33–33.01)	0.253 NS	1.40 (0.44–4.47)	0.566 NS
>10	1		1	
8–10	7.78 (0.84–72.00)	0.071 NS	6.53 (0.66–65.10)	0.109 NS
<8	3.62 (0.40–33.01)	0.253 NS	1.19 (0.16–8.63)	0.863 NS
WBC				
>15	1		1	
10–15	0.59 (0.10–3.33)	0.549 NS	1.50 (0.32–7.09)	0.609 NS
<10	2.22 (0.45–10.99)	0.329 NS	0.59 (0.10–3.53)	0.567 NS
Arm				
Group 0	1		1	
Group 1	14.40 (2.11–98.47)	**0.007 S**	27.44 (4.63–162.76)	**<0.001 S**
Group 2	30.79 (4.51–210.06)	**<0.001 S**	58.05 (6.51–517.68)	**<0.001 S**

CRP, C-reactive protein; CI, confidence interval; h, hours; Hb, haemoglobin; HR, hazard ratio; NS, not significant; S, significant; VAS, visual analogue score; WBC, white blood cell; *p*, *p* value with significance set at less than 0.05. Bold values imply that the difference of severe crisis elimination observed between group 1 and group 0, and group 2 and group 0 is statistically significant.

## Data Availability

The data sets used and/or analyzed during the current study are available from the corresponding author on reasonable request.
